# 

**Published:** 1872-04-15

**Authors:** 


AMONG the advertisements in this number, we would call the attention of our readers especially to those of E. H. Sargent; Bliss & Torrey; and Codman & Shurtleff. We think the instruments described in the latter are the best of their kind in use.

Money Receipts to May 1st, 1872.—Dr. J. Priestman, $3.00; Aug. Rhoads, $3.00; L. S. Rogers, $5.00; M. H. Cushing, $3.00; S. G. Claybourg, $3.00; W. T. Knapp, $1.50; Hewett & Dysart, $3.00; W. W. Wynne, $9.00; H. W. Smith, $3.00; H. Rosbough, $3.00; A. C. Simonton, $3.00; A. H. Thompson, $3.00; G. W. Rohr, $6.00; G. W. Phillips, $9.00; W. H. Mussey, $3.00; D. B. Trimble, $3.00; D. E. Thayer, $6.00; S. Hemminway, $5.00.

